# The brain in oral clefting: preliminary results of a systematic review with meta-analyses

**DOI:** 10.1192/j.eurpsy.2022.1644

**Published:** 2022-09-01

**Authors:** K.A. Sándor-Bajusz, A. Saadi, E. Varga, G. Csábi, G. Antonoglou, S. Lohner

**Affiliations:** 1 University of Pécs, Paediatrics, Pécs, Hungary; 2 Borlänge Specialist Clinic, Region Dalarna, Adult Psychiatric Division, Borlänge, Sweden; 3 University of Pécs, Department Of Paediatrics, Pécs, Hungary; 4 Medical University of Graz, Oral Surgery Department, Faculty Of Dentistry, Graz, Austria

**Keywords:** cleft lip, cleft palate, Brain, Neuroimaging

## Abstract

**Introduction:**

Previous neuroimaging studies of individuals with nonsyndromic oral clefts have revealed subtle brain structural differences compared to matched controls. Additional studies strongly suggest that the higher incidence of neuropsychiatric issues observed in these individuals may be explained by these neuroanatomical differences. Currently there are no studies that have assessed the overall empirical evidence of the effect of oral clefts on the brain.

**Objectives:**

Our aim was to summarize available evidence on potential brain structure differences in individuals with nonsyndromic oral clefts and their matched controls. In the current presentation, we discuss the results of regional brain structural differences.

**Methods:**

Five databases were systematically searched in September 2020 for case-control studies that reported neuroimaging in healthy individuals and individuals with nonsyndromic oral clefts. Duplicate study selection, data extraction, random effects meta-analyses of mean differences (MDs) and their 95% confidence intervals were performed in order to compare regional brain MRI volumes.

**Results:**

We have identified 245 records following the database searches, from which 12 records met the inclusion criteria. Quantitative data on brain structure were available in three studies.The cerebellum, occipital and temporal lobes were significantly smaller in the cleft group compared to controls (MD: -12.46, 95% CI: -18.26, -6.67, n=3 studies; MD:-7.39, 95% CI: -12.80, -1.99, n=2 studies; MD: -10.53, 95% CI: -18.23, -2.82, n=2 studies, respectively).

**Conclusions:**

There may be structural brain differences between individuals with nonsyndromic oral clefts and their controls based on the available evidence.

**Disclosure:**

No significant relationships.

